# Designing and Using Digital Mental Health Interventions for Older Adults: Being Aware of Digital Inequality

**DOI:** 10.3389/fpsyt.2019.00568

**Published:** 2019-08-09

**Authors:** Alexander Seifert, Dominique Alexandra Reinwand, Anna Schlomann

**Affiliations:** ^1^University Research Priority Program “Dynamics of Healthy Aging”, University of Zurich, Zurich, Switzerland; ^2^Center of Gerontology, University of Zurich, Zurich, Switzerland; ^3^Rehabilitative Gerontology, Faculty of Human Sciences, University of Cologne, Cologne, Germany

**Keywords:** mobile intervention, gerontechnology, seniors, digital divide, mental health

## Background

Worldwide, approximately 15% of the people older than 60 suffer from a mental disorder, such as depression and anxiety disorders (1). A total of 322 million people worldwide are affected. The prevalence rates vary by age and peaks in older adults (2). Modern devices, such as smartphones and tablets, can be used to provide digital interventions for various health-related issues. Digital interventions are promising in their ability to provide researchers, medical practitioners, and patients with a dynamic and individualizable tool for assessing behavior and behavioral change, consultation, treatment, and integrated care. These digital interventions can help patients manage their diseases or their general health as a form of disease prevention. This is particularly important in older people, as individuals often have to deal with highly complex interactions involved in managing their daily lives along with the consequences of a multitude of chronic diseases (3). Digital interventions can, for example, assess, control, and positively influence mental health and well-being among older patients (4). Particularly with regard to mental health, digital mental health interventions (DMHIs) appear to close a gap in healthcare provision. Many patients with mental health problems have to wait a long time to get an appointment for initial counseling or therapy; in rural areas, older patients may face long travel distances, and many people are still afraid of stigmatization and avoid therapy completely (5). Reviews and meta-analyses have shown the benefits of DMHIs, for example, for people with depression and anxiety (6–8).

While DMHIs are becoming more important and popular, there exists a danger that older people will be excluded. When not comfortable with new technologies, older adults can experience barriers to accessing DMHIs, which might result in larger healthcare inequalities (9). This can happen if only already-advantaged populations use and benefit from these interventions. Therefore, this article will outline and discuss the problems in this field and make recommendations for future developments.

## Digital Divide Across Age Groups

Internet access and the usage of Internet-connected devices, such as smartphones, are becoming more widespread globally, which paves the way for DMHIs. Nevertheless, a digital gap between generations remains; older adults make less frequent use of the Internet or smartphones than do younger adults (10, 11). For example, in the United States, 67% of people 65 and older have access the Internet, whereas nearly all younger people are online (12). A representative survey across Switzerland and 16 European Union countries showed that only 49% of people aged 50 and older use the Internet (13). The study indicated that Internet use among older adults is predicted by personal factors such as age, gender, education, income, health, prior experience with technology, social salience (Internet use among the members of one’s social network), and contextual factors, such as country-specific wealth and communication technology infrastructure.

Against this background, older adults are at risk of being excluded from DMHIs for the following five reasons: first, from the perspective of environmental gerontology, new technologies may contribute to a stimulating environment for successful aging (14), but older people often lack experience, skills, social support, and access to digital tools, and they may face numerous barriers to the effective use of these technologies (15). This could increase the risk that older adults perceive their digital environment as exclusionary rather than stimulating (16). Second, as mentioned above, a considerable number of older adults do not use digital technologies. Third, older, retired adults do not need to use new technologies as part of their jobs, which might reduce their motivation to adopt new technologies in their spare time. Fourth, besides an individual’s age, it is also important to consider his or her level of technological socialization (17); the baby-boom generation, for instance, did not grow up with digital technologies and, therefore, they have not been socialized to use them. Finally, from a developmental perspective, people become more vulnerable as they grow older. Therefore, they have to make a greater effort to learn to use new technologies and often have to overcome the barriers arising from having fewer cognitive, physical, financial, and social resources (18).

## Can All Older Adults Benefit From DMHIs?

Despite the fact that older people use the Internet or mobile devices, such as smartphones, less frequently than the rest of the population, there is an interest among older adults in integrating new technologies into their healthcare (19). Reviews have found that DMHIs are effective in reducing depressive symptoms and stress experiences (3, 20). Nevertheless, in a study of 121 people from community mental health services, Ennis et al. (21) found that lack of skills is the reason older patients do not engage with computers or mobile devices and are, therefore, less frequent users of DMHIs. The available digital interventions are often not designed for frail and technologically unskilled older adults or for their age-related learning and usability needs (22).

While it has been reported that older participants appreciated the intervention (23), it must be mentioned that older people who dropped out of the intervention program were not included in such evaluations. Dropout rates are known to be high in Web-based interventions (24), but it has been shown that older people are more motivated to use digital health interventions than younger people (25). However, there is a lack of evaluation regarding systematical noninclusion of technical unskilled older adults within DMHIs. We assume a bias regarding inclusion of technically skilled older adults and noninclusion of technically unskilled older adults among empirical evaluation studies of digital interventions.

Research has noted that, next to the difference of access or non-access (which is known as the fist digital divide), Internet skills vary within the older age group with the oldest less skilled than the youngest (the second level digital divide) (10). Furthermore, there is a third digital divide that affects the outcomes and benefits of digital health intervention usage (26). Due to less access and less usage, older adults gain fewer benefits of those interventions. It seems that older adults have a higher risk of being more disadvantaged on all three levels than younger digital health intervention users.

Furthermore, only a few studies have focused on older participants’ satisfaction and perceived usefulness of digital interventions. It is known that only a minority of interventions (8%) considered the specific needs of older people during the development and design process (23), so it can be assumed that most digital interventions are designed for advanced users and neglect unskilled older people. These tendencies have been described as the innovativeness-needs paradox, which means that individuals who objectively need a given innovation the most are the ones least likely to adopt it (27).

## Recommendations

Given the rapid expansion of digital interventions, it seems worthwhile to educate older adults in how to use DMHIs that could be useful in their daily lives. It would be helpful to offer support and training to these people to increase their self-efficacy and digital literacy skills. Learning new technological skills can even foster a certain sense of competence and autonomy (28) that can encourage the efficient use of a digital intervention. These learning tools can be generally provided by adult educational services, such as senior universities or adult education centers, but should especially be offered prior to intervention participation through the provider of the intervention. The special learning needs of older adults need to be considered in these educational services (29), with attention paid to things such as the tempo of the learning session and the technological skill background of the older participants.

In the literature, there is an assumption that learning tools will alone suffice to increase the use of DMHI, but this is hard to prove because there are only a few studies available that have tested the effect of training on intervention usage and outcomes (30). A scoping review, however, suggests that usability and technical problems, lack of value of an intervention, and insufficient training are among the most important barriers facing older adults in using digital interventions (19). These findings suggest that in the future, in order to reduce age-related inequalities in digital interventions, more should be invested in training, education, and support to increase participation among older adults and decrease dropout rates.

Older adults often use technology selectively and in unexpected ways. They often develop their own digital skills and strategies. Research on aging an technology should consider this usage behavior as legitimate and not as mistakes or wrong usage because it helps to understand the role of technology in older adults’ everyday lives (31). Therefore, it is crucial to motivate developers and professional users (e.g., researchers, medical practitioners, and companies within the health sector) of DMHIs to take a closer look at how different designs and content can be tailored in a way that encourages trust and facilitates use among older people. This requires a development of DMHIs tools (software and hardware) that include the needs of older users (15, 29). It is known that taking end users into account increases the usage and effectiveness of interventions (32). For example, Darvishy et al. (33) developed a brochure for age-appropriate mobile applications that is grounded in the recommendations of the W3C Web Accessibility Initiative (34) and focuses on older adults’ needs for an accessible and useful application. The applications should, for example, be presented in a clear, intuitive, self-explanatory, transparent, and consistent way, and navigation within the application should be logical, with steps communicated clearly and kept to a minimum.

Therefore, it is beneficial to involve older adults as part of participative research before developing a new digital intervention. After developing such an intervention, it is also crucial to invest time in educating the participants regarding all aspects of the intervention before they begin to use it. Furthermore, during the intervention, a reachable support hotline and contact partner can be used to assist the older participants when needed. Finally, after an intervention, the daily challenges in using it as reported by the participants can be used as important input for further development and indicator for sustained long-term use.

## Conclusions

DMHIs offer unique and innovative opportunities for older adults with mental disorders, such as depression or anxiety. Developers and medical practitioners in the mental health field can use the advantages of digital tools to provide older adults with a helpful instrument. However, the approach also brings challenges, especially when technologically unskilled older adults cannot benefit from DMHIs because of their lack of access or digital skills. On the one hand, researchers must be aware that findings of the usefulness, acceptance, and effectiveness of DMHIs might be very limited and biased when certain groups of older adults are not included. On the other hand, older adults are not generally technology-averse and already use a number of technologies as part of their everyday life; although sometimes differently than younger generations. Developers, practitioners, and researchers in this field must be aware of this digital inequality and provide training tools and support services, in addition to developing digital interventions that consider the background knowledge and needs of older people, who are not “digital natives” (35).

## Author Contributions

All authors provided substantial contributions to this article from conception to final approval and share the same opinion.

## Conflict of Interest Statement

The authors declare that the research was conducted in the absence of any commercial or financial relationships that could be construed as a potential conflict of interest.
